# Les tumeurs glomiques de la main: une étude rétrospective de 11 cas

**DOI:** 10.11604/pamj.2016.24.262.8154

**Published:** 2016-07-20

**Authors:** Hassan Boussakri, Abdelhalim Elibrahimi, Chafik Hahem, Mohammed Bachiri, Nawal Hammas, Mohammed Elidrissi, Mohamed Shimi, Jean Luc Roux, Abdelmajid Elmrini

**Affiliations:** 1 Service de Chirurgie Osteoarticulaire (B4), CHU Hassan II, Faculté de Médecine et de Pharmacie Sidi Mohammed Ben Abdullah, 30000 Fès, Maroc; 2 Institut Montpelliérain de la Main, clinique climentville, 34000 Montpellier, France; 3 Laboratoire d’Anatomie Pathologie, CHU Hassan II Faculté de Médecine et de Pharmacie Sidi Mohammed Ben Abdullah, Fès, Maroc

**Keywords:** Tumeur, glomique, main, Tumour, glomic, hand

## Abstract

Tumeur glomique selon MASSON est une prolifération neuro-myo-arterielle bénigne. Elle représente environ 1% -5% de toutes les tumeurs de la main. La douleur est le signe clinique principal. Le diagnostic de certitude repose sur un faisceau d’arguments: clinique et radiologique, mais seul l’histologie qui permet la confirmation. Une série de 11 patientsa été revue rétrospectivement, l’âge moyen était de 36,27ans avec un recul moyen de 34,40 mois, et des extrêmes de 8 et 48 ans. L’exérèse chirurgicale étaitréalisée chez tous les patients. Cette stratégie nous a permis d’obtenir des résultats satisfaisants.

## Abstract (en)

MASSON described glomus tumour as a benign neuromyoarterial proliferation. It represents approximately 1% -5% of all tumors of the hand. Pain is the main clinical sign. Definitive diagnosis is based on a body of evidence: clinical and radiological but only histology will allow confirmation. We conducted a retrospective study of 11 patient whose average age was of 36, 27 years (range 8-48 years), with a mean follow-up period of 34,40 months. All patients underwent surgical excision. This strategy allowed us to achieve satisfactory results.

## Introduction

Les tumeurs glomiques de la main se définies comme une hyperplasie de l’appareil glomique des doigts qui se situe sous la peau, caractérisé par la présence d’une anastomose artério-veineusequi contrôle la température locale. Masson [1] en 1924 était le premier à définir cette entité pathologique sous la ‘tumeur neuro-myo-arteriel. Ces tumeurs sont relativement rares, Elles représentent environ 1%-5% de toutes les tumeurs de la main [2]. Bien qu’elles peuvent se développer dans toute partie du corps, la localisation du membre supérieure est souvent au niveau des extrémités dans la région sous-unguéales [3]. Elles se manifestent cliniquement par des douleurs lancinantes paroxystiques dans la partie distale des doigts avec une intolérance au froid [4]. Les auteurs rapportent une etude rétrospective monocentrique concernant 11cas de tumeur glomiquede la main sur une période de sept ans entre février 2009 et juin 2015. Le but de cette étude est d’analyser les donnéesépidémiologiques, le profil clinique ainsi qu’insister sur l’intérêt de traitement chirurgicale enfin discuter les résultats de notre série.

## Méthodes

**Figure 1:**
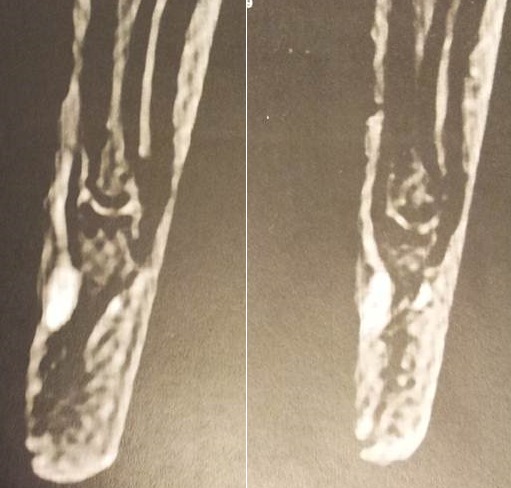
Aspect IRM d’une tumeur glomique

IL s’agit d’une étude rétrospective monocentriqueconcernant11 cas du patients présentant une tumeur glomique de la main, diagnostiqué et confirmé par une etude anatomopathologique sur une périodeseptans, entre février 2009 et juin 2015 colligé au service de chirurgie osteoarticulaire B4 Fès Maroc (Tableau 1). Le critère d’inclusion se référait à l’existence d’une douleur au niveau de la main aggravé par une pression au bout des doigts, avec intolérance au froid. Le diagnostic de la tumeur glomique est basé sur un faisceau d’argument clinique, radiologique notamment les données d’échographie et d’IRM (Figure 1) et confirmé par une étude anatomopathologique. Un seul patient a été pris en charge initialement dans un deuxième hôpital et nous avons exigé de récupéré le dossier médical initial. Ont été exclues les patients présentant un autre tableau clinique similaire type syndrome de Raynaud, les cas avec une discordance entre la clinique et radiologie et d’autre part les données anatomopathologique, en fin les dossiers inexploitables et les malades perdus de vu. Nous avons demandez systématiquement des radiographies standard de la main face et profil qui ont été normaux. L’échographie était réalisée chez 3 patients. L’imagerie par résonance magnétique (IRM) a été effectuée dans tous les cas (Tableau 1, Figure 1). Par contre aucun cas la tomodensitométrie (TDM).

**Tableau 1:** La série

Patient	âge	sexe	doigt	imagerie
1.	35	F	2^eme^ doigt	RX ;echo;IRM
2.	28	F	3^eme^ doigt	RX ;IRM
3.	43	H	2^eme^ doigt	RX ;echo;IRM
4.	24	F	2^eme^ doigt	RX ;IRM
5.	37	F	5^eme^ doigt	RX ;IRM
6.	54	F	1^er^ doigt	RX ;echo;IRM
7.	27	F	2^eme^ doigt	RX ;IRM
8.	42	H	3^eme^ doigt	RX ;IRM
9.	39	F	2^eme^ doigt	RX ;IRM
10.	41	F	3^eme^ doigt	RX ;IRM
11.	29	F	2^eme^ doigt	RX ;IRM

**Figure 2:**
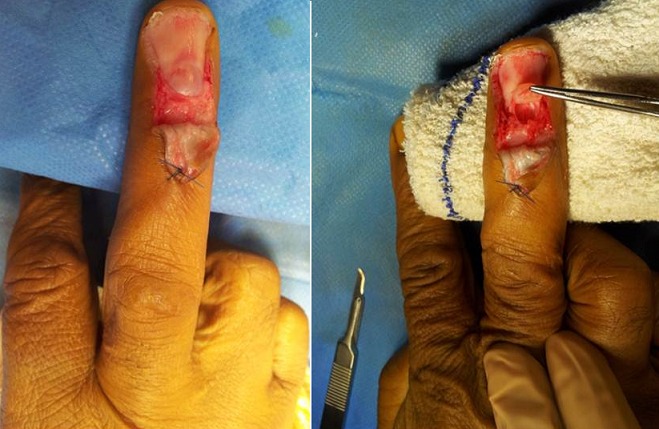
La voie d’abord chirurgicaletrans-unguéale

**Figure 3:**
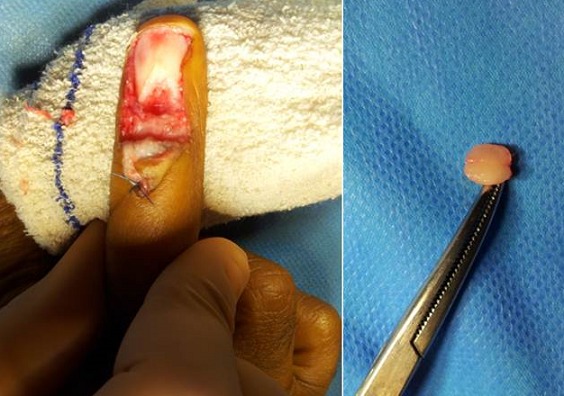
Découverte d’une tumeur encapsulée

**Figure 4:**
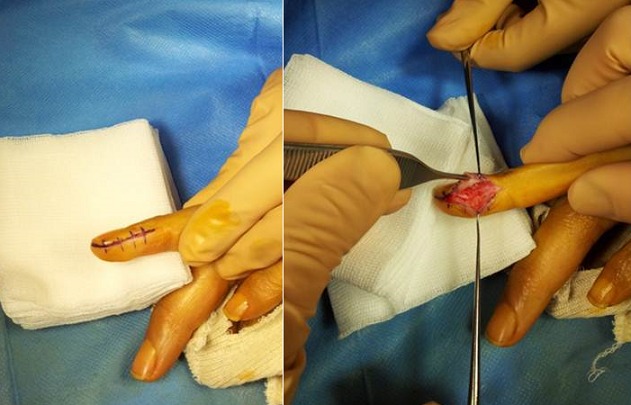
La voie d’abord chirurgicalelatérale

**Technique opératoire:** Les indications opératoires sont basés sur les données de l’examen cliniques et surtout ceux d’imagerie notamment IRM. Le patient était installé en décubitus dorsal, le membre supérieur concerné dans le champ opératoire. Sous anesthésie locorégionalun garrot à la racine du membre. La voie d’abord utilisée étaittrans-unguéal dans 9 cas (Figure 2, Figure 3) et dans 2c as la voie d’abord était latérale (Figure 4). En principe la dissection de la tumeur est faiteà l’aide avec loupes grossissantes 2,5×. Une tumeur relativement bien définie est accouché (Figure 2, Figure 3) La suture de lit de l’ongle a été faite en utilisant prolene 7-0, et l’ongle a été repositionné après avoir été fermée avec du nylon 4-0. Tous les patients ont été revus cliniquement avec une évaluation de la douleur sur échelle analogique [5] et le questionnaire dequick DASH [6].

## Résultats

Tous les patients ont été opérer par deux chirurgiens seniors spécialistes en chirurgie du membre supérieur pour une tumeur glomique diagnostiquer. Parmi ces 11 patients (Tableau 1), il y avait 09 Femmes et 02 Hommes dont l’âge moyen était de 36,27ans, avec des extrêmes de 24 et 54 ans. Un patient avait été pris initialement en charge dans un autre hôpital. Sur le plan clinique tous les patients de notre série étaient douloureux avec sur échelle analogique une moyenne de 6/10 (EV:4 à 10) [5]. Les tumeurs qui ont été retirées étaient circulaire, arrondie, encapsulé, mesurant environ 4 à 10 mm (Figure 4). Le recul moyen était de 34,30 mois, avec des extrêmes de 8 et 48 mois, un écart type de 11,22, une médiane de 33. Sur le plan anatomopathologique, toutes les tumeurs étaient bien limitées, encapsulé avec des zones fibreuses. Sous grossissement microscopique on note la présence des cellules épithélioïdes, régulières, entouré des vaisseaux sanguins dilatés. A forte grossissement, les cellules épithélioïdes présente un noyau rondqui n’avait presque aucune mitose observés. Les résultats thérapeutiques étaient satisfaisants. Chez tous les patients on a noté une disparition de la douleur avec un score de quick DASH chiffré à 5 par contre on a noté deux cas de déformation modérer de l’ongle sans retentissement fonctionnelle, ni esthétique.

## Discussion

Les tumeurs glomiques se produisentsur uneprolifération des tissus du corps de glomus. Cette entité anatomique est riche en anastomose artério-veineuse spécialisée, chargée de la thermorégulation. Ils se développent dans plusieurs sites, ainsi que des localisations ont été rapportées dans les muqueuses ou dans autres organes tels que l’estomac, les poumons, la trachée, et les os [7, 8]. Le site de prédilection pour les tumeurs glomique est la main particulièrement la région sous-unguéale, la face latérale des doigts [9]. Les tumeurs glomiques se manifestent essentiellement par une douleur paroxystique. L’aggravation des symptômes par le froid est évocatrice du diagnostic. D’autres symptômes en faveur, une coloration bleuâtre sous-unguéale, l’hypoesthésie. Plusieurs hypothèses etiopathogéniques ont été proposées pour comprendre la physiopathologie de la douleur dans les tumeurs glomiques: leurs capsules les rendent sensibles à la pression, la présence de mastocytes au sein des tumeurs glomiques est à l’origine d’une libération de substances telles que: l’héparine, la 5-hydroxytryptamine, l’histamine, responsable d’une stimulation des récepteurs de la pression et du froid [10], aussi la présence d’une innervation de la tumeur est responsable de la douleur [11]. Le diagnostic de tumeur glomique des doigts doit être évoqué en se basant sur un faisceau d’arguments. Cependant, les signes cliniques ne sont pas pathognomonique, surtout devant la localisation profonde et le diamètre petit de la tumeur.la confirmation est basé sur imagerie et notamment résultats d’anatomopathologie. Les tests diagnostiques sont nombreux, le test de la pression, le test de Hildreth est effectuée, le membre supérieure doit être exsangue, ensuite un garrot pneumatique est gonflé à 250 mm Hg, le test est positif quand on relâcher le brassard provoque une douleur soudaine et intense et le test de la sensibilité au froidpositif par immersion de la main dans l’eau froide [12]. Cesqui peuventguider le diagnostic mais leur sensibilité est limité [3–9]. Concernant les diagnostics différentielles d’une tumeur glomique, un certains nombre de diagnostic doivent être évoqué notamment, les fibromes, neuropathiques, l’arthrite et les névralgies [11–13].

Des examens complémentaires sont nécessaires pour étayer le diagnostic. La radiographiestandard est généralement normale mais son intérêt principal pour éliminer les autres diagnostics différentiels qui peut engendrer une lésion osseuse. L’échographie est le meilleur examen paraclinique pour guider le diagnostic d’une tumeur glomique mais aussi suivre l’évolution [14]. Cet examen reste operateur dépendant ce qui nécessite un radiologue spécialisé en imagerie osteoarticulaire, mais aussi un matériel adapté (sonde) notammentpour détecté la lésionfacilement [15]. IRM est un examen non invasif de choix pour mettre en évidence une tumeur glomique et discuté les diagnostics différentiels au stade radiologique. Ainsi que, à l’IRM, une tumeur glomique est décrite comme une lésion légèrement hypo-intense en T1, qui prend le produit de contraste après injection de gadolinium et hyper-intense en T2 [16, 17]. Exérèse chirurgicale complète de la tumeur glomiquereste le traitementchoix, elle permet d’obtenir l’indolence et éviter la récidive par une exérèse totale de la tumeur et son environnement [3–9], dans les cas où les limites de la tumeur ne sont pas claire en raison de l’hémorragie, l’exérèse totale de la tumeur est souvent impossible et la possibilité de récidive tumoral est probable [18]. Concernant les voies d’abord chirurgicale, l’abord trans-unguéal est avantageuse car permet une l’excision complète de la tumeur siégeant sur la face dorsale du lit de l’ongle, mais certains inconvénients sont décrit noté, notamment la qualité de l’onglesi le lit n’est pas soigneusement suturé [19], sur ce point l’abord latéral trouve son intérêt par une tumorectomie sous périostée sans endommagé l’ongle, mais certains complication possible notamment une lésion des nerfs digitaux [20]. Concernant la récidive tumorale, habituellement causée par une résection incomplète de la tumeur initiale. Afin d’éviter une exérèse incomplète, une bonne exposition chirurgicale doit être la règle et assurée une bonne voie d’abord chirurgicale de choix et en fin un curetage osseux est utile afin d’éliminer le reliquat du tissus tumoral. Les tumeurs glomiques sont classées soit en unique ou multiple, selon leur présentation clinique. Tumeurs glomiques uniques sont beaucoup plus fréquentes que la variante multiple. Selon Rettig et Strickland [21], la variante unique se trouve principalement dans la main, avec 25% à 75% se produisent dans la région sous-unguéale. Tumeurs multiples glomiques sont indolores en général et de développer chez les jeunes enfants ou les hommes dans un mode autosomique dominant [22]. Les tumeurs glomiques sont constitués de cellules glomiques, des vaisseaux sanguins et des muscles lisses et sont classées en trois catégories en fonction de leur composition: glomangiome présente un aspect d’hémangiomecaverneux, tumeur glomique solideprésente un diagnostic différentiel avec une tumeur solide épithéliale et glomangiomyome, il reprend l’architecture d’une tumeur glomique solide ou d’une glomangiome mais on note une prédominance des muscles lisses. Bien que les tumeurs glomiques soient essentiellement bénignes, la transformation sarcomateuse de cette tumeur est exceptionnels [23].

## Conclusion

L’orthopédiste doit pas oublier la possible de présence d’une tumeur glomique au niveau de la main, responsable d’une douleur chronique avec un échec de diagnostic précis. Cette tumeur doit donc être évoquée devant toute douleur isolée des doigts. L’exérèse chirurgicale est une solution efficace.

### Etat des connaissances actuelles sur le sujet

- Diagnostic initial difficile: elle passe inaperçu;
- Pathologie mystérieuse.

### Contribution de notre étude à la connaissance

- Première étude marocaine et africaine qui discute cette pathologie;
- une pathologie plus moins fréquente dans la population maghrébine, mais sous diagnostiqué;
- Echographie est un examen clef.
